# Foot gangrene following Tagraxofusp treatment for blastic plasmacytoid dendritic cell neoplasm: Case report

**DOI:** 10.1002/jha2.541

**Published:** 2022-08-04

**Authors:** Jad Sibai, RuiQi Chen, Ibrahim Al Nabhani, Maria Agustina Perusini, Hassan Sibai

**Affiliations:** ^1^ Division of Medical Oncology and Hematology, Princess Margaret Cancer Centre University Health Network Toronto Canada

**Keywords:** blastic plasmacytoid dendritic cell neoplasm, case report, gangrene, Tagraxofusp

## Abstract

Blastic plasmacytoid dendritic cell neoplasm (BPDCN) is a rare and aggressive hematologic malignancy. It is associated with poor prognosis and heterogenous presentation. The CD123‐directed cytotoxin, Tagraxofusp, is a targeted therapy for BPDCN. Here, we report an 81‐year‐old female diagnosed with BPDCN. The patient was treated with Tagraxofusp and underwent a remarkably long remission (>20 months) without stem‐cell transplantation. She, however, experienced blue toe syndrome and left foot gangrene. We postulate that these previously unreported side effects were caused by microembolization. Characterization of the incidence of thrombo‐ and microembolizations in such a context, as well as prophylactic management options, are warranted.

## 1 INTRODUCTION

Blastic plasmacytoid dendritic cell neoplasm (BPDCN) is an uncommon and aggressive hematologic malignancy, often presenting as cutaneous lesions with or without bone marrow (BM) involvement [1]. Its incidence rate is 4.5 per 10,000,000 [2]. Tagraxofusp is an approved treatment for BPDCN patients older than 2 years [3]. It is a recombinant fusion protein made of human interleukin‐3 (IL‐3) conjugated to a truncated diphtheria toxin. The structural composition of Tagraxofusp allows it to bind the IL‐3 receptor α (CD123) present on cancerous cells [4].

**FIGURE 1:**
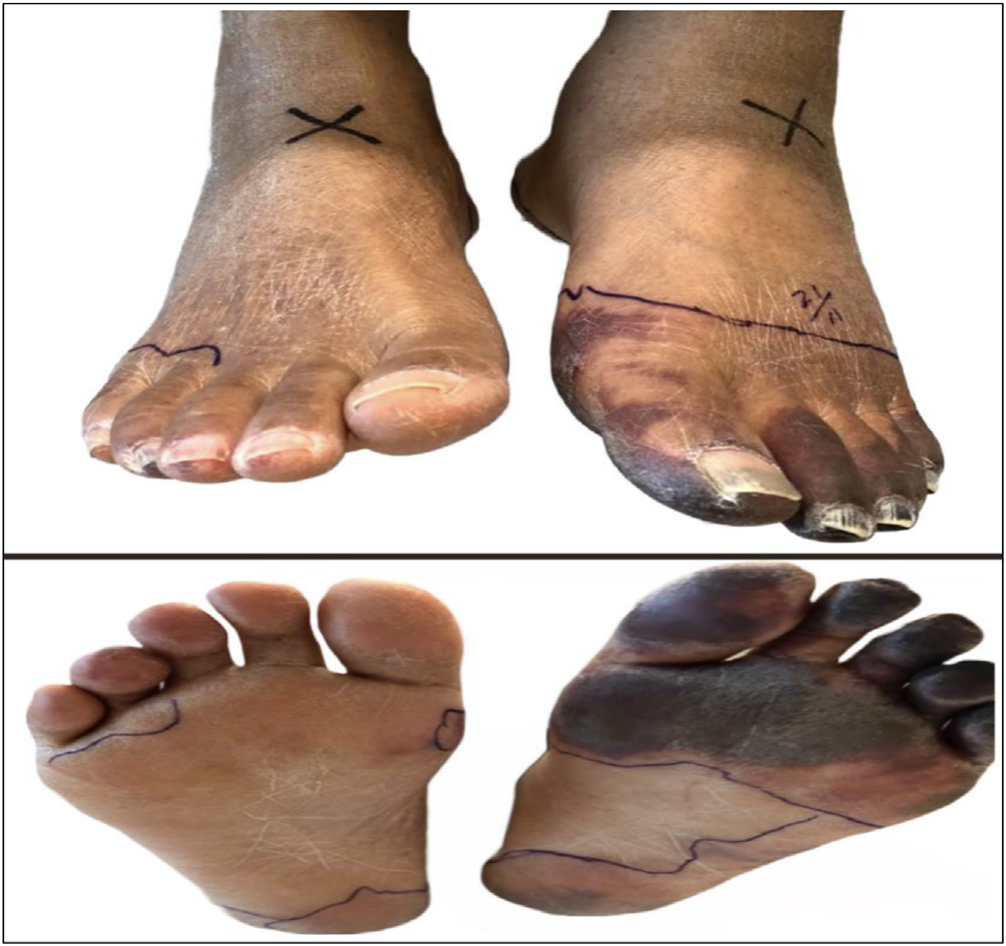
Dorsal (top) and plantar (bottom) view of feet 12 days post‐Tagraxofusp induction. Gangrene pronounced across the five left foot digits and hallux/plantar aspects (right side)

The side effect profile of Tagraxofusp is notable for capillary leak syndrome (CLS), which has been reported in over 50% of cases [3]. Other complications include hepatotoxicity, anemia, hypersensitivity, edema, hypoalbuminemia, thrombocytopenia, and transaminitis [5, 6]. We are, however, reporting the first case of Tagraxofusp‐linked blue toe syndrome (BTS) followed by peripheral gangrene in a patient with long‐lasting remission from BPDCN (Figure 1).

## 2 CASE REPORT

An 81‐year‐old female presented with multiple skin lesions and a skin biopsy consistent with BPDCN. BM aspirate and biopsy demonstrated BM involvement. Flow cytometry was positive for BPDCN. Next‐generation sequencing of the skin biopsy revealed mutations in ASXL1, IDH2, and KRAS. Her complete blood count, lactate dehydrogenase, renal function, and liver profile were all within normal limits. She had a previous stroke, hypothyroidism, hypertension, and type 2 diabetes mellitus.

She was treated with Tagraxofusp for 5 days, ensuring that albumin was >35 g/L via regular infusions. On day 10 post‐induction, she became bicytopenic with hemoglobin at 86 g/dl and platelet count at 20 × 10^9^/L. She experienced hypoxia, tachycardia, volume overload and weight gain, leading to a presumed diagnosis of CLS necessitating an intensive care unit (ICU) transfer for monitoring. Within a few hours of the ICU transfer, bilateral toe discolorations consistent with BTS were observed. Vascular surgery was consulted, with peripheral arterial and venous Doppler ultrasounds unrevealing for overt thromboembolisms, suggestive of a possible microembolization event. Antiplatelets and anticoagulation could not be administered immediately given her thrombocytopenia (platelet count at 7 × 10^9^/L). At the onset of her symptoms, she was treated with intravenous methylprednisone, which unfortunately resulted in no improvement in toe discoloration. After partial platelet recovery on day 18 of the treatment, she was started on aspirin and low‐molecular‐weight heparin.

**FIGURE 2:**
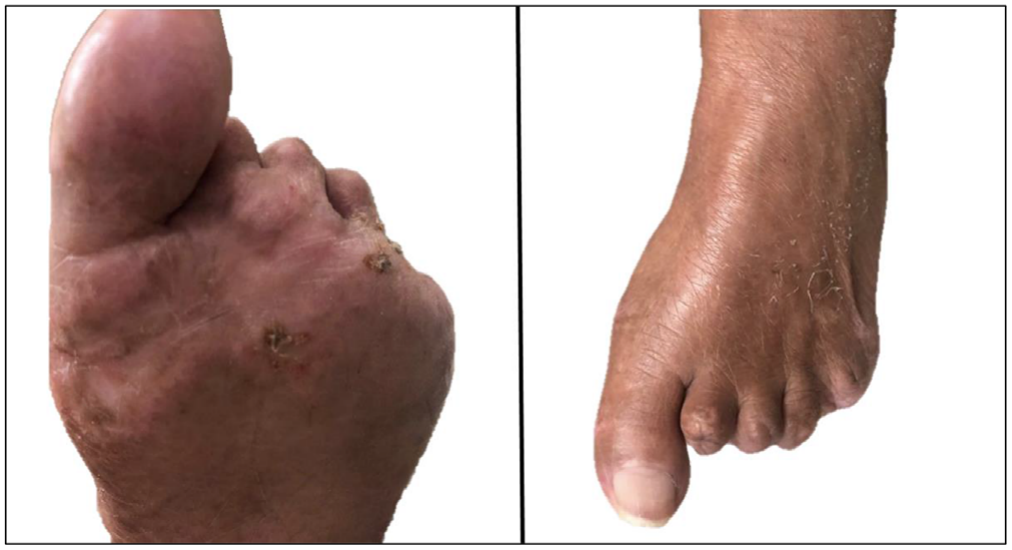
Plantar (left) and dorsal (right) views of the left foot following amputation of digits 2–5 due to irreversible necrotic tissue damage. The left foot is no longer gangrenous

With combined antiplatelet and anticoagulation therapies, the patient's right foot completely recovered, while her left foot improved in the hallux and plantar aspects. Unfortunately, the left foot's digits 2–5 remained gangrenous. Given progressive tissue necrosis, these digits were amputated (Figure 2). Computed tomography scan of the chest and abdomen revealed no apparent blood clots. Subsequent diagnostic tests (for vasculitis, connective tissue disease, disseminated intravascular coagulation, echocardiogram) also revealed no abnormalities. Despite these complications, she achieved complete remission (CR) after only one Tagraxofusp induction cycle, confirmed via a negative repeat BM biopsy and aspirate and the resolution of her primary skin lesions. A Tagraxofusp consolidation cycle was attempted 4 months later, where she again experienced fatigue, CLS, hypoxia, and severe bicytopenia, resulting in treatment termination after two doses. Fortunately, there has been no documented BPDCN relapse; she has now been in CR for 20 months.

## 3 DISCUSSION

This case report highlights two significant findings. It introduces previously unreported Tagraxofusp toxicities—the presentation of BTS and peripheral foot gangrene. On the other hand, it presents a long‐lasting CR from BPDCN after only one successful Tagraxofusp cycle. Thus, while the drug did lead to unintended consequences necessitating amputation, this treatment was successful in eliminating BPDCN, emphasizing a high chemotherapeutic efficacy.

A multicohort study by Pemmaraju et al. [5] revealed that among a subgroup of 32 previously untreated BPDCN patients, the median duration of Tagraxofusp exposure was 96 days (range: 2–927). Additionally, the patients underwent a median of five treatment cycles (range: 1–43). Of this population, only one patient reported a CR longer than 18 months without receiving stem‐cell transplantation. As such, the reported case, in both its significantly short exposure to Tagraxofusp (one completed cycle; 7 days exposure) and long CR of 20 months, implies that the reported toxicities did not undermine the drug's antitumor effect, which resulted in a longer than average remission.

Reporting the toxicities of Tagraxofusp will help inform potential prophylactic strategies. Of the regularly reported toxicities, CLS was experienced by the patient. We postulate that it is perhaps the result of off‐target CD123 binding and subsequent neutralization of endothelial cells. The intravenous drug generally induces apoptotic cell death via diphtheria toxin‐mediated inactivation of elongation factor 2 (EF2), impeding downstream protein translation pathways. The toxin is endocytosed upon IL‐3 binding to CD123, translocating into the cytosol where it mediates the ADP ribosylation of EF2, inactivating it [7]. Although CD123 is overexpressed among the cancer cell population, it is also found on the surface of endothelial cells, possibly predisposing an off‐target apoptotic effect of Tagraxofusp on blood vessel walls, resulting in systemic plasma leakage to surrounding regions and by‐extension CLS [8]. Vascular insults are known for triggering hemostasis (and thrombosis), rendering them potentially relevant to our case report.

Nonetheless, the diagnosis of CLS in the reported patient is probable but not certain, given some inconsistent symptoms. For instance, she only gained 4 kg (baseline weight: 57 kg) during the treatment cycle (5 days; 5 kg+ expected), experiencing mild fluid overload. She also remained hemodynamically stable, citing no hypotension (>90 mmHg systolic pressure) or hypoalbuminemia while maintaining a normal ejection fraction.

The cause of BTS is likely multifactorial. CLS alone does not account for asymmetrical foot gangrene (Figure 1). As such, a likely cause of BTS is microembolization, which may have led to acro‐ischemia and gangrene at the toes [9]. Such microthrombi would have likely developed from a drug‐induced pro‐coagulation state [10]. The complete recovery of the right foot and the partial recovery of the left foot (hallux and digit 1) following the administration of anticoagulants can be the result of uneven microthrombi dislodgement. Since microembolization was a likely contributing mechanism, the administration of pretreatment prophylaxis, such as aspirin and anticoagulants, may reduce the relative risk of both thrombo‐ and microembolization. Further characterizations of the incidence of thrombo‐ and microembolizations in such a context, as well as effective preventative and management options, are warranted.

## AUTHOR CONTRIBUTIONS

Jad Sibai wrote the paper. Dr. RuiQi Chen, Dr. Maria Agustina Perusini, Dr. Ibrahim Al Nabhani, and Dr. Hassan Sibai critically edited and added relevant information to the paper. Dr. Hassan Sibai proposed the report and provided invaluable patient details.

## CONFLICT OF INTEREST

The authors declare they have no conflicts of interest.

## FUNDING INFORMATION

The authors received no specific funding for this work.

## ETHICS STATEMENT

Consent for the publication and academic discussion of the case was obtained from the patient.

## PATIENT CONSENT STATEMENT

Informed consent was obtained.

## Data Availability

The data that support the findings of this study are available on request from the corresponding author. The data are not publicly available due to privacy or ethical restrictions.
